# Older Adults’ Subjective Age as a Potential Psychological Resource in Clinical Management of Chronic Illnesses

**DOI:** 10.1093/geroni/igaa057.2816

**Published:** 2020-12-16

**Authors:** Anyah Prasad, Nidya Velasco Roldan, Meghan Hendricksen, Natalie Shellito

**Affiliations:** 1 University of Massachusetts Boston, Boston, Massachusetts, United States; 2 University of Massachusetts, Boston, Boston, Massachusetts, United States

## Abstract

Subject age is predictive of future morbidity and mortality and can be potentially viewed as a psychological resource. However, there seems to be a reciprocal relationship between subjective age and health. In a series of analyses, we demonstrated that various measures of health status such as number of chronic illnesses, self-rated health and sensory impairment have an adverse association with older adults’ subjective age. Specifically, chronic illnesses seem to have a period effect and age effect. Living with chronic illness over a period of time seems to attenuate its association with subjective age. Similarly, the association between chronic illnesses and subjective age gets weaker with increase in older adults’ chronological age. Therefore, asking those living with chronic health conditions and specifically younger older adults about their subjective age and providing appropriate resources, counseling and reassurance about chronic illness management may prevent the downstream negative health effects of increased subjective age.

